# GY-SLAM: A Dense Semantic SLAM System for Plant Factory Transport Robots

**DOI:** 10.3390/s24051374

**Published:** 2024-02-20

**Authors:** Xiaolin Xie, Yibo Qin, Zhihong Zhang, Zixiang Yan, Hang Jin, Man Xu, Cheng Zhang

**Affiliations:** 1 Longmen Laboratory, Luoyang 471003, China; xiexiaolin@haust.edu.cn; 2 College of Agricultural Equipment Engineering, Henan University of Science and Technology, Luoyang 471003, China; lyzzh@haust.edu.cn (Z.Z.); 210321041670@stu.haust.edu.cn (Z.Y.); 220320261796@stu.haust.edu.cn (H.J.); 220320261812@stu.haust.edu.cn (M.X.); 230320261490@stu.haust.edu.cn (C.Z.)

**Keywords:** SLAM, YOLOv5, GhostNet, octree maps, grid maps, plant factory

## Abstract

Simultaneous Localization and Mapping (SLAM), as one of the core technologies in intelligent robotics, has gained substantial attention in recent years. Addressing the limitations of SLAM systems in dynamic environments, this research proposes a system specifically designed for plant factory transportation environments, named GY-SLAM. GY-SLAM incorporates a lightweight target detection network, GY, based on YOLOv5, which utilizes GhostNet as the backbone network. This integration is further enhanced with CoordConv coordinate convolution, CARAFE up-sampling operators, and the SE attention mechanism, leading to simultaneous improvements in detection accuracy and model complexity reduction. While mAP@0.5 increased by 0.514% to 95.364, the model simultaneously reduced the number of parameters by 43.976%, computational cost by 46.488%, and model size by 41.752%. Additionally, the system constructs pure static octree maps and grid maps. Tests conducted on the TUM dataset and a proprietary dataset demonstrate that GY-SLAM significantly outperforms ORB-SLAM3 in dynamic scenarios in terms of system localization accuracy and robustness. It shows a remarkable 92.59% improvement in RMSE for Absolute Trajectory Error (ATE), along with a 93.11% improvement in RMSE for the translational drift of Relative Pose Error (RPE) and a 92.89% improvement in RMSE for the rotational drift of RPE. Compared to YOLOv5s, the GY model brings a 41.5944% improvement in detection speed and a 17.7975% increase in SLAM operation speed to the system, indicating strong competitiveness and real-time capabilities. These results validate the effectiveness of GY-SLAM in dynamic environments and provide substantial support for the automation of logistics tasks by robots in specific contexts.

## 1. Introduction

Simultaneous Localization and Mapping (SLAM) is one of the key technologies in the field of robotic navigation, enabling robots to accurately determine their position and create maps of their surroundings without any prior information [1]. Particularly in the field of mobile robotics, Visual SLAM [2] (VSLAM) has become the focus of research and application due to its cost-effectiveness and its ability to provide rich environmental information [3]. However, most existing VSLAM algorithms are based on the assumption of a static environment [4]. In dynamic environments, when extracting features from dynamic targets, especially those with strong texture information, it may lead to increased trajectory errors or even tracking loss [5]. Therefore, in the process of transferring vegetable packages from the stacking area to the pre-cooling area in plant factory transportation robots, the SLAM system is affected by dynamic targets such as humans and collaborative robots. This necessitates a SLAM system that can detect and eliminate dynamic feature points in real time to enhance system accuracy and robustness [6].

Semantic SLAM, produced by the fusion of deep learning and SLAM, provides a promising solution. It can predict the dynamic characteristics of predefined targets and provide the system with functional attributes and semantic information about them. This not only enhances the accuracy of robot localization in dynamic scenarios but also lays the foundation for autonomous intelligent path planning and advanced handling tasks. RGB-D cameras, which provide precise depth information through physical measurements, can also be employed for target detection and image segmentation [7]. However, while image segmentation can reduce the interference of dynamic targets, it comes at the cost of system real-time performance [8]. In light of this, YOLO (You Only Look Once) single-stage target detection networks, known for their compact size and efficient real-time performance, have become an ideal choice. With improvements, they can achieve positioning accuracy close to that of image segmentation SLAM while maintaining significantly higher real-time performance, thus striking a balance between SLAM system accuracy and real-time capabilities [9]. Currently, some advanced SLAM systems based on semantic segmentation, such as RDS-SLAM [10], build upon ORB-SLAM3 by introducing a dedicated semantic thread and a semantics-based optimization thread. These threads run in parallel with others, allowing the tracking thread to proceed without waiting for semantic information, theoretically achieving real-time tracking in dynamic environments. On the other hand, target detection-based SLAM systems, like YG-SLAM [11], incorporate a GPU-accelerated YOLOv5 object detection module. Combining the results of object detection with the LK optical flow method during the dynamic feature point removal stage significantly enhances recognition speed.

In this paper, we propose a novel real-time dense semantic SLAM system named GY-SLAM, specifically designed for plant factory transportation robots. This system integrates deep learning techniques to assist robots in perceiving the environment from both semantic and geometric perspectives. GY-SLAM can not only effectively identify and eliminate feature points on predefined dynamic targets but also construct a pure static dense point cloud and generate an octree map and a grid map for navigation, which improves the positioning and mapping capabilities of the SLAM system in dynamic scenes. The main contributions of this paper include the following:

1. Based on ORB-SLAM3, dense mapping, target detection threads, and a dynamic feature elimination module have been added. A method for constructing dense point clouds based on statistical filtering and voxel down-sampling has been proposed, resulting in the generation of octree maps and grid maps.
2. A target detection dataset containing various robots, humans, and vegetable packages was created. Additionally, a SLAM dataset containing RGB and depth information, ground truth trajectories, and the aforementioned targets were collected.
3. A lightweight target detection model named GY, based on YOLOv5s, was developed with lightweight processing by incorporating GhostNet. CoordConv coordinate convolution, CARAFE up-sampling operators, and SE attention mechanisms were introduced into the model.
4. The above GY model and the enhanced SLAM system are successfully integrated into a GY-SLAM visual-dense semantic system and evaluated.

The remaining structure of this paper is as follows: Section 2 reviews relevant work by other scholars in the field. Section 3 provides a detailed introduction to the framework and proposed methods of GY-SLAM. Section 4 describes the materials and methods used in this research. Section 5 reports the experimental evaluation results on our proprietary dataset and the TUM RGB-D dataset. Section 6 discusses the major findings of this research. Section 7 summarizes the research achievements of this paper and outlines directions for future work.

## 2. Related Work

The robustness of SLAM systems in dynamic environments has become a focal point of research for numerous investigators. The primary challenge is how to effectively detect and eliminate dynamic features and avoid using feature points extracted from moving objects for positioning and mapping [12]. As research has progressed, many excellent algorithms have endeavored to incorporate target detection and image segmentation techniques from deep learning into the SLAM system, providing essential semantic priors for detecting and eliminating dynamic feature points [13].

Li et al. [14] fused RGB-D camera and encoder information, utilizing the SegNet image segmentation network based on Caffe to segment moving objects in images. The DS-SLAM system proposed by Yu et al. [15] passes images with per-pixel semantic labels to the tracking thread through the SegNet image segmentation thread, thus separating outlier points belonging to dynamic targets. Bescos et al. [16] proposed the DynaSLAM algorithm, which leverages Mask R-CNN to obtain images with per-pixel image segmentation and instance labels for dynamic target detection. Ren et al. [17] presented the VI-MID system, which employs Mask R-CNN to extract object masks and relies on rendering masks obtained from object-level maps for continuous tracking of targets. However, per-pixel image segmentation methods such as SegNet and Mask R-CNN, while achieving high classification accuracy, are slow in speed, which does not meet the real-time target detection requirements for robots. Target detection methods based on bounding boxes exhibit significantly higher efficiency compared to per-pixel image segmentation methods.

Zhang et al. [18] integrated modules for target detection and recognition using YOLO into the RGB-D SLAM framework, building semantic octree maps based on object-level entities. Zhang et al. [19] augmented the ORB-SLAM2 system with a YOLOv5-based object detection and recognition module, achieving real-time and rapid detection of dynamic features. Guan et al. [20] incorporated a YOLOv5 target detection module into the tracking module of ORB-SLAM3 and generated static environment point cloud maps using RGB-D cameras. Wang et al. [21] proposed YPD-SLAM, a system based on Yolo-FastestV2 target detection and CAPE plane extraction, capable of running on the CPU while maintaining relatively high detection accuracy. Song et al. [22] introduced YF-SLAM, which utilizes the lightweight target detection network YOLO-FastestV2 to provide semantic information in dynamic environments for ORB-SLAM2. Wu et al. [23] presented YOLO-SLAM, which improved detection speed by replacing darknet-53 with darknet-19 for target detection. Liu et al. [24] introduced Dynamic-VINS, which utilizes YOLOv3 to detect various dynamic elements on resource-constrained mobile platforms.

When the dynamic objects in the environment are known in advance, the use of deep learning methods can be highly effective, but these methods are heavily reliant on the quality of the network [25]. Simple network architectures may not effectively recognize objects in certain situations, while complex architectures may slow down system performance. This challenge has driven researchers to seek lightweight and efficient yet stable target detection models to enhance the quality of SLAM systems. This demand provides clear direction and reference for our work on lightweight and improvements.

## 3. Improved System Description

In this section, we will provide a detailed explanation of our proposed GY-SLAM system. This system combines lightweight deep learning techniques with advanced strategies for enhancing target detection networks, effectively achieving the functionalities of target detection and dynamic feature elimination. Furthermore, GY-SLAM possesses the capability to construct precise, dense maps, laying a solid foundation for the accurate localization, path planning, and transportation tasks of robots in the dynamic environment of plant factories. We will now proceed to introduce the implementation details of each key component, starting with the overall framework of the system.

### 3.1. Overview of the GY-SLAM System

**Figure 1:**
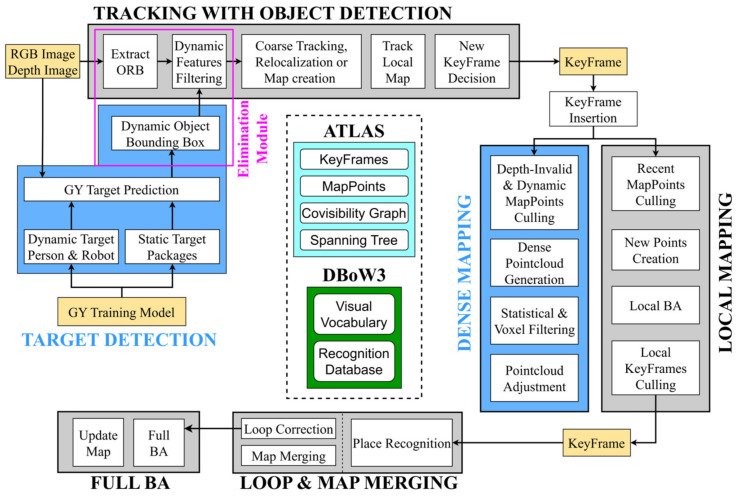
GY-SLAM System Framework.

The framework of the GY-SLAM system proposed in this paper is illustrated in Figure 1. The system comprises five main threads running in parallel: Tracking, Local Mapping, Loop and Map Merging, Target Detection, and Dense Mapping. Among these, the Target Detection and Dense Mapping threads represent innovative extensions based on ORB-SLAM3, while the Local Mapping and Loop and Map Merging threads remain consistent with ORB-SLAM3.

#### 3.1.1. ORB-SLAM3

ORB-SLAM3 is the first feature-based SLAM system that supports monocular, stereo, and RGB-D cameras. It is capable of visual, visual-inertial SLAM, and multi-map creation [26]. The system effectively utilizes short-term, medium-term, long-term, and multi-map data association, thereby effectively suppressing drift and ensuring high-precision localization in medium to large loop-closure scenarios. This comprehensive data association capability significantly improves the system’s adaptability and stability, which enables it to achieve a localization accuracy of up to 9 mm.

#### 3.1.2. Dynamic Feature Elimination

We first collected a dataset of YOLO images containing elements relevant to the plant factory transport robot work. Subsequently, we trained the GY target detection model using the GY network. In GY-SLAM, the GY model serves as input to provide predefined target information to the Target Detection Thread.

The Target Detection Thread is responsible for processing the video stream captured by the camera frame by frame. After inferring and analyzing the images using the GY model to identify predefined targets and generate bounding boxes for them, it outputs semantic information, localization information, and confidence to the Dynamic Feature Elimination Module in the Tracking Thread. Within the Tracking Thread, we have embedded a Dynamic Feature Elimination Module that receives the output from the Target Detection Thread. After extracting ORB feature information in the Tracking Thread, this module eliminates feature points within the dynamic area. This ensures that only static feature points are used for subsequent pose estimation and mapping.

#### 3.1.3. Dense Mapping

While ORB-SLAM3 is effective, the sparse maps it generates cannot be directly used for robot path planning and navigation. Therefore, constructing a pure static, dense map that can be used for navigation is crucial for transport robots. In the Dense Mapping Thread, after the system receives keyframes from the Tracking Thread, it first performs eligibility filtering on map points to obtain a basic, pure static dense point cloud. This process includes removing map points with significant errors based on effective camera depth, eliminating outliers based on outlier marking, and removing dynamic feature points based on dynamic target localization information provided by the Target Detection Thread. The final result is a relatively stable, pure static, dense point cloud.

In constructing the 3D octree map, statistical filtering is used to remove outlier map points in the dense point cloud, which is achieved by calculating the average distance between each point and the points within its surrounding neighborhood. Assuming that the calculation results follow a Gaussian distribution, outlier points with unqualified average distances are filtered out based on the standard deviation. Subsequently, the point cloud density is reduced by voxel down-sampling technology. This technique divides three-dimensional space into uniform voxels, samples only one central point in each voxel as a representative, and assigns the points in each voxel to the octree structure. Through recursive operations, we can obtain the octree map. The octree map not only reduces computational load but also preserves critical geometric structures, making it suitable for robot modeling and navigation in complex, dynamic environments.

Grid maps play a crucial role in robot collision detection, navigation, and path planning. To construct a grid map, we first analyze the robot’s obstacle clearance height and working height. Then, we project the dense point cloud within this height range onto a grid. After filtering and dilation processing, we obtain a two-dimensional grid map.

### 3.2. Overview of the GY Lightweight Target Detection Network

The YOLOv5s [27] is adopted as the foundation, and through lightweight and a series of improvements, the lightweight GY target detection network is built, aiming to balance accuracy and computing resources while maintaining high-speed performance.

**Figure 2:**
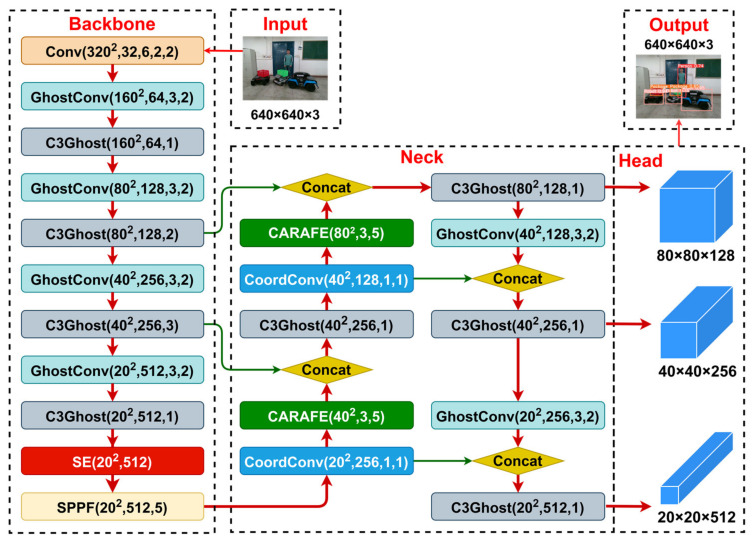
GY network architecture.

In this article, the lightweight GhostNet network is integrated with the YOLOv5s, and then three improvements are conducted to enhance model accuracy and generalization. Firstly, CoordConv coordinate convolution is introduced in the FPN structure, enabling the model to perceive the positional information of feature image pixels. Secondly, the CARAFE up-sampling operator is introduced to expand the receptive field, allowing the network to perform up-sampling based on the semantic information from the input feature maps. Finally, at the end of the Backbone, the SE channel attention mechanism is introduced to focus on global feature maps, effectively modeling the interdependence between channels. The resulting GY network architecture is illustrated in Figure 2.

#### 3.2.1. GhostNet Neural Network

GhostNet [28] is a lightweight and efficient CNN network proposed by Huawei Noah’s Ark Lab in 2020. Its Ghost module first generates intrinsic feature maps using fewer convolutional kernels and then produces many ghost feature maps through a series of cost-effective linear transformations. These ghost feature maps are capable of extracting the desired information from the intrinsic features. In terms of efficiency and accuracy, the lightweight GhostNet reduces model complexity, making it particularly suitable for mobile robots with limited memory and computing resources. The computational cost of Ghost convolution compared to regular convolution is as follows:

(1) $$\mathrm{cost}\;1=h^{\prime }\times w^{\prime }\times n\times k\times k\times c$$

(2) $$\mathrm{cost}\;2=h^{\prime }\times w^{\prime }\times \frac{n}{s}\times k\times k\times c+(s-1)\times h^{\prime }\times w^{\prime }\times \frac{n}{s}\times k\times k$$

where cost 1 denotes the computational cost of the regular convolution, cost 2 denotes the computational cost of the Ghost convolution, $h^{\prime }\times w^{\prime }\times c$ denotes the height, width, and number of channels of the output feature maps, k denotes the convolution kernel size, and s denotes the number of ghost feature maps generated by each intrinsic feature map. Since s≪c, the theoretical acceleration ratio $r_{s}$ of using the Ghost convolution to replace the regular convolution can be approximated as follows:

(3) $$r_{s}=\frac{\mathrm{cost}\;1}{\mathrm{cost}\;2}\approx \frac{s+c}{s+c-1}\approx s$$

#### 3.2.2. CoordConv Coordinate Convolution

CoordConv [29] is a coordinate convolution module proposed by Uber in 2018. Traditional convolutions only capture local information when the convolution kernel performs local operations and do not know the spatial location of the current convolution kernel. CoordConv adds two additional channels into the input feature map of convolution to represent pixel coordinates, enabling the network to learn complete translation invariance or a certain degree of translation dependency according to different task requirements. Simultaneously, it allows the convolution to perceive feature spatial information to some extent during learning, thereby enhancing detection accuracy and robustness.

#### 3.2.3. CARAFE Up-Sampling Operator

CARAFE [30] is a lightweight up-sampling operator proposed by Wang et al. in 2019. It can aggregate contextual information over a large receptive field and supports instance-specific content-aware processing, dynamically generating adaptive up-sampling kernels. During CARAFE computation, the Kernel Prediction Module is responsible for perceiving the content at each target location and generating a reassembled kernel. The Content-Aware Reassembly Module uses the predicted kernel to reassemble the features, increasing the emphasis on information from relevant feature points in local regions. The reassembled feature map contains more semantic information compared to the original feature map.

#### 3.2.4. SE Attention Mechanism

**Figure 3:**
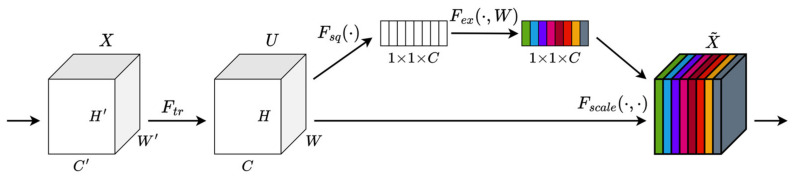
A Squeeze-and-Excitation block.

SE [31] is a channel attention module proposed by Hu et al. in 2019. The SE module models the relationship between channels by introducing a Squeeze operation and an Excitation operation. In the Squeeze stage, it compresses the output feature map of the convolutional layer into a feature vector through a global average pooling operation. Then, in the Excitation stage, the weight vector of a channel is learned by using the fully connected layer and the nonlinear activation function. This weight vector is applied to each channel on the original feature map to weigh the features of different channels. In this way, the SE module can adaptively learn the importance of each channel and adjust the channel contribution in the feature map according to the needs of the task. This attention mechanism helps the network better focus on important feature channels, thereby improving model performance. The structure of the SE building block is illustrated in Figure 3.

## 4. Equipment and Methods

In this research, considering the need for robots to recognize three elements: humans, robots, and vegetable packages, a new SLAM dataset was collected. This dataset serves as a practical platform for testing the SLAM algorithms of plant factory transport robots. Two separate systems on a single server were used for GY deep learning model training and SLAM algorithm testing. The experimental environment configuration is detailed in Table 1, and the left side of the combination of the two parameters is the deep learning configuration parameter.

### 4.1. GY Model Training

Our YOLO image dataset primarily consists of images captured by the Intel RealSense Depth Camera D455 with an aspect ratio of 4:3. Additionally, the dataset includes human images from open datasets and various robot and vegetable package images downloaded online. We carefully selected a total of 955 images, resized them proportionally to a width of 640 pixels, and annotated them using the Labelimg tool. The classification labels include Person, Robot, and Package. Following the principles of data augmentation, we augmented the dataset by a factor of three, resulting in a total of 2865 images to enhance the model’s generalization capability. Our proprietary dataset has universal adaptability to other network models. The ratio of the training and validation datasets was set to 8:2, while the test dataset consisted of video streams captured by the GY-SLAM system. The hyperparameter configuration of GY network training is shown in Table 2.

### 4.2. GY-SLAM Dataset Acquisition

**Figure 4:**
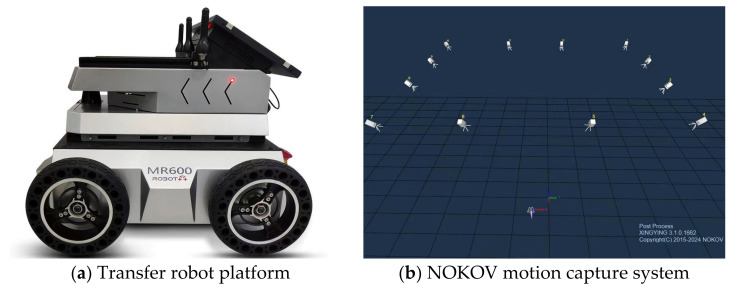
Equipment for collecting the GY-SLAM dataset. (**a**) MR600 mobile robot, D455 camera, and reflective markers; (**b**) 12 NOKOV Mars 2H cameras and motion capture system.

We used the D455 camera to capture RGB and depth data and employed the NOKOV Motion Capture System to obtain real-time trajectory ground truth for the robot. The MR600 transport robot from ShiHe Company served as the mobile platform, with the D455 camera mounted on a bracket at the top of the robot. We incorporated the work elements that the transport robot faced into the dataset to validate the subsequent target detection network’s ability to recognize targets and eliminate dynamic feature points. The dataset encompasses various scenarios, including handheld and wheeled robot shooting, fast and slow motions, as well as normal and multi-rotational scenarios. The equipment used for collecting the SLAM dataset is shown in Figure 4, with specific parameters provided in Table 3.

## 5. Experimental Results

### 5.1. GY Experimental Results

In this article, while ensuring model detection accuracy and FPS exceeding 30, we prioritized reducing the complexity of the GY model to minimize computational resource consumption during inference. We utilized metrics including the mean Average Precision at the IoU threshold of 0.5 (mAP@0.5), the number of model parameters (Parameters), the computational complexity measured in Giga Floating-Point Operations Per Second (GFLOPs), and the model size (Weight) as evaluation criteria. The latter three metrics, to some extent, reflect the model’s complexity.

#### 5.1.1. Lightweight Network Comparative Experiment

In this experiment, we used YOLOv5s as the baseline model and integrated it with three mainstream lightweight feature extraction networks for comparative experiments in order to obtain the most cost-effective lightweight network. The results are shown in Table 4.

The results presented in Table 4 reveal that substituting the original CSPDarkNet53 backbone feature extraction network in YOLOv5s with various lightweight networks significantly reduced the model’s parameters, computation, and size. However, this substantial reduction in complexity was accompanied by varying degrees of decreased detection accuracy. When integrated with ShuffleNetV2, the model exhibited the smallest reduction in complexity but underwent the largest decrease in mAP@0.5, which was 4.901%. In contrast, integration with MobileNetV3 led to the most substantial reduction in complexity, along with a decrease in mAP@0.5 of 3.492%. Upon combining with GhostNet, the reduction in the model’s complexity was intermediate compared to the other two models, with the smallest decline in mAP@0.5 recorded at 0.669%. Consequently, the network GY*, resulting from the combination of GhostNet and YOLOv5s, was selected as the optimal original lightweight network.

#### 5.1.2. Ablation Experiment

To validate the contribution of the improved methods proposed in this study to the model performance, we designed an ablation experiment based on YOLOv5s as a benchmark, with the results presented in Table 5.

Based on the results in Table 5 and using the GY* lightweight network from test 2 as a reference, the following conclusions were drawn from comparative tests: In test 3, the introduction of the CoordConv convolution module in the FPN structure of the Neck part added 2.116% in parameters, 1.218% in computation, and 2.003% in weight, but resulted in a 0.972% increase in mAP@0.5. In test 4, incorporating the CARAFE up-sampling operator led to an additional 3.806% in parameters, 3.343% in computation, and 3.738% in weight, with a 1.039% improvement in mAP@0.5. Test 5, which combined both the CoordConv and CARAFE, resulted in an increase of 5.922% in parameters, 4.574% in computation, 5.741% in weight, and a 1.136% enhancement in mAP@0.5. Test 6, which introduced the SE channel attention module at the end of the Backbone part, added 0.890% to the parameters, 0.336% to the computation, and 0.935% to the weight, while increasing the mAP@0.5 by 0.691%. Test 7, combining both the CoordConv and SE, led to an additional 3.006% in parameters, 1.554% in computation, and 2.804% in weight, but raised the mAP@0.5 by 1.057%. In test 8, the GY model was developed by integrating the GhostNet lightweight network, CoordConv convolution module, CARAFE up-sampling operator, and SE attention module. Compared to the original GY* lightweight model, although there was a 6.812% increase in parameters, a 4.909% increase in computation, and a 6.542% increase in weight, there was also a noTable 1.183% improvement in mAP@0.5. In comparison with the original YOLOv5s model, the GY model exhibited a 43.976% reduction in parameters, a 46.488% reduction in computation, and a 41.752% reduction in weight, while simultaneously achieving a 0.514% increase in mAP@0.5, reaching 95.364%.

The results indicate that the GY model, developed by enhancing YOLOv5s, not only significantly reduces model complexity but also boosts average detection accuracy, consequently making the model’s performance superior.

#### 5.1.3. Attention Mechanism Comparative Experiment

To validate the superiority of the introduced SE attention module, we used the original lightweight network GY* as the baseline and conducted comparative experiments by replacing it with four different attention mechanisms: CBAM, CA, ECA, and EMA. The results are presented in Table 6.

The data in Table 6 clearly illustrates that the increase in model complexity is remarkably minimal, regardless of the type of attention module introduced. Interestingly, the introduction of CBAM and ECA modules actually led to a decrease in the model’s mAP@0.5, contrary to expectations of an increase. Among the attention modules that did enhance average detection accuracy, the EMA module, despite being the most complex, ironically resulted in the least improvement in mAP@0.5, a mere increase of 0.049%. Both the CA and SE modules induced almost identical increments in model complexity. However, the CA module improved the model’s mAP@0.5 by only 0.464%, which was less effective compared to the SE module. Significantly, our results demonstrate that the SE module, which we proposed, achieves the highest enhancement in mAP@0.5 of 0.691% among all the models tested.

#### 5.1.4. Algorithm Comparative Experiment

In order to verify the superior performance of our proposed GY network, we conducted comparative experiments with other target detection algorithms, and the results are shown in Table 7.

**Figure 5:**
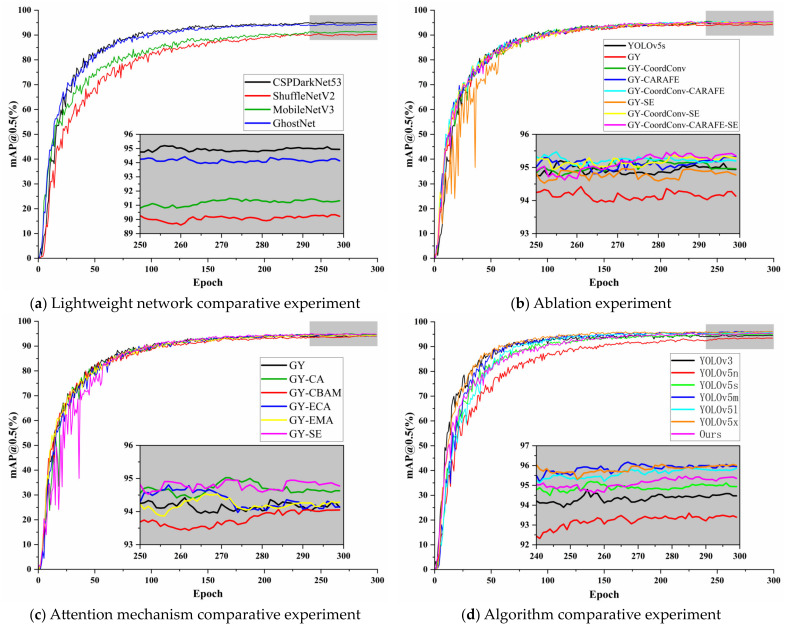
The graph of mAP@0.5 curve. (**a**) The mAP@0.5 curves for different models in the lightweight network comparative experiment; (**b**) The mAP@0.5 curves for different models in the ablation experiment; (**c**) The mAP@0.5 curves for different models in the attention mechanism comparative experiment; (**d**) The mAP@0.5 curves for different models in the algorithm comparative experiment.

The results presented in Table 7 illustrate that the model developed with our innovative GY network exhibits unparalleled cost-effectiveness. It significantly surpasses the smaller YOLOv5n, achieving a 1.998% increase in mAP@0.5. When compared with larger models such as YOLOv5m, l, x, and YOLOv3, the GY model makes a modest trade-off in average detection accuracy, yet it benefits from a marked reduction in complexity—decreasing by a factor of 5 to 20 times. The mAP@0.5 curves for various models across different experiments are illustrated in Figure 5.

**Figure 6:**
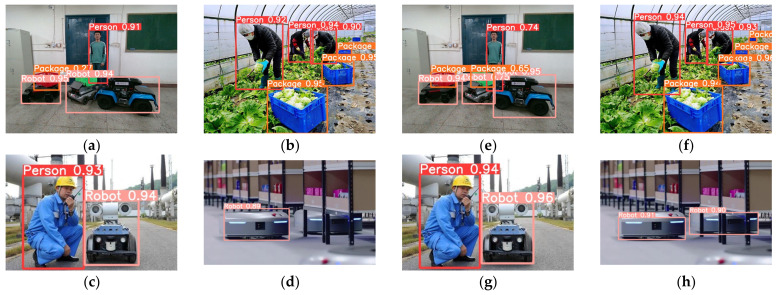
Comparison graph of detection result between YOLOv5s and GY. The images (**a**–**d**) on the left side represent the detection results of YOLOv5s in four images; The images (**e**–**h**) on the right side represent the detection results of GY in four images same with YOLOv5s.

From Figure 5, it can be observed that the improvement strategies we chose at different stages are relatively optimal. We compared the detection effectiveness of the GY model with the YOLOv5s model. The detection results are shown in Figure 6, where the GY model is capable of identifying small and occluded targets, and its overall detection accuracy is also higher than that of the YOLOv5s network.

### 5.2. GY-SLAM Experimental Results

We integrated the GY model into our GY-SLAM system for target recognition tasks. The performance of GY-SLAM was evaluated on both our proprietary dataset and the TUM RGB-D dataset, with an assessment of the tracking time consumption. Additionally, based on ORB-SLAM3, we evaluated the performance improvement of the GY-SLAM system and the performance of DynaSLAM and DS-SLAM. Absolute Trajectory Error (ATE) and Relative Pose Error (RPE) were commonly used to evaluate the quality of visual SLAM systems, where ATE is suitable for measuring the global consistency of a trajectory, while RPE is more appropriate for assessing drift in translation and rotation. We utilized Root Mean Square Error (RMSE) and Mean Error (Mean) to reflect ATE and RPE as evaluation indicators. Each algorithm was executed 10 times in the same sequence, and the average of these 10 results was taken as the indicator’s value.

#### 5.2.1. Performance Evaluation on the TUM RGB-D Dataset

The comparative results of different algorithms on various dynamic sequences of the TUM RGB-D dataset are presented in Table 8, Table 9 and Table 10. Table 8, Table 9 and Table 10 clearly demonstrate that GY-SLAM shows significant improvements in ATE and RPE compared to ORB-SLAM3. In the ATE results of Table 8, under high-dynamic scenarios, RMSE and Mean are enhanced by up to 92.5864% and 93.6967%, respectively. In low-dynamic scenarios, such as in the Fr3_s_static sequence, the improvements in RMSE and Mean are 17.3077% and 19.3548%, respectively. It is noted that in low-dynamic scenes, DynaSLAM and DS-SLAM slightly outperform GY-SLAM. This is due to their ability to further differentiate static features within dynamic regions, whereas GY-SLAM eliminates all features in these areas, leading to a scarcity of features available for tracking. The translational and rotational drift results in RPE, as shown in Table 9 and Table 10, exhibit a similar trend and magnitude of error reduction as seen with ATE.

**Figure 7:**
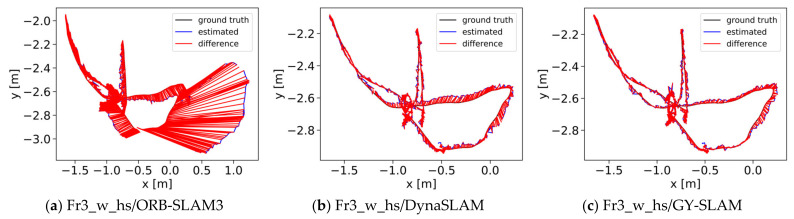

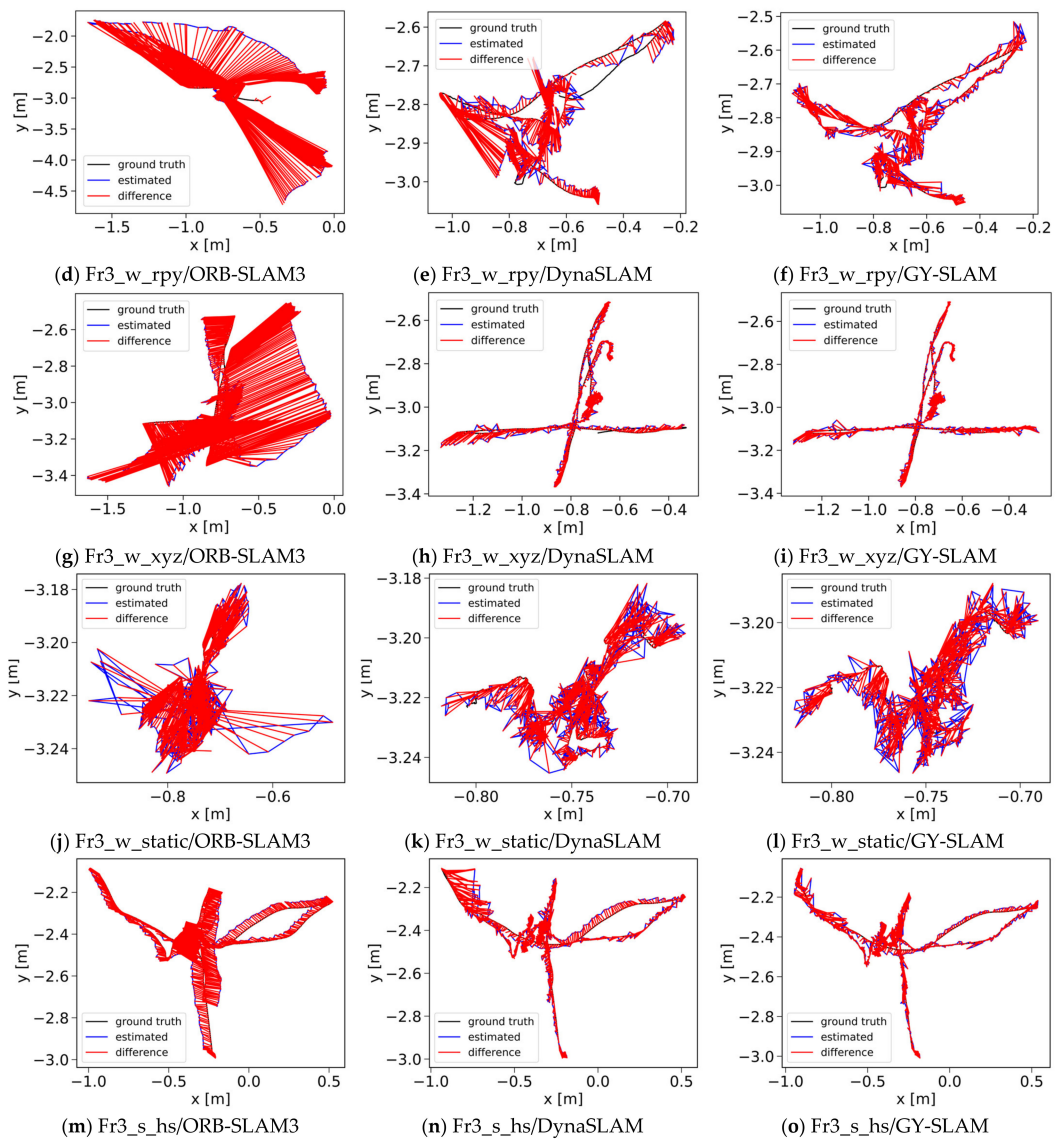
Absolute trajectory error diagram. (**a**) Images (**a**–**c**), respectively, represent the ATE graphs of ORB-SLAM3, DynaSLAM, and GY-SLAM on the Fr3_w_hs sequence; (**b**) Images (**d**–**f**) represent the ATE graphs of the three algorithms on the Fr3_w_rpy sequence; (**c**) Images (**g**–**i**) represent the ATE graphs of the three algorithms on the Fr3_w_xyz sequence; (**d**) Images (**j**–**l**) represent the ATE graphs of the three algorithms on the Fr3_w_static sequence; (**e**) Images (**m**–**o**) represent the ATE graphs of the three algorithms on the Fr3_s_hs sequence.

The results indicate that the absolute trajectory error of GY-SLAM has been reduced by approximately an order of magnitude compared to ORB-SLAM3, achieving centimeter-level or even millimeter-level precision. This improvement is attributed to the semantic information generated by GY, which effectively assists the system in identifying and eliminating dynamic feature points. Compared with DynaSLAM and DS-SLAM, GY-SLAM shows better performance on some sequences. The system performs well in high-dynamic scenarios but is slightly constrained in low-dynamic environments. Figure 7 shows the Absolute Trajectory Error (ATE) graphs for ORB-SLAM3, DynaSLAM, and GY-SLAM on partial sequences. As can be seen from Figure 7, the error in GY-SLAM is significantly reduced.

#### 5.2.2. Performance Evaluation on the Proprietary Dataset

Table 11 reveals that GY-SLAM has significantly improved the system’s performance in terms of ATE, with the maximum improvements in RMSE and Mean reaching as high as 28.0829% and 28.4339%, respectively. Meanwhile, we noted differences in the magnitude of improvement across various tests: test 2 demonstrated a higher increase compared to test 3, possibly due to the sudden starts and stops of the robot in test 3, which led to accuracy degradation. The greater improvement in test 5 over test 6 could be attributed to the white wall in test 6, which hindered the extraction of sufficient feature points for stable tracking. The more significant improvement in test 5 compared to test 4 is speculated to result from the GY target detection network’s effective identification and handling of dynamic feature points in the high-dynamic scenarios of test 5. The increase in test 5 over test 1, and generally larger improvements in tests 4–6 compared to tests 1–3 might be due to the rapid movement of the robot causing visual blurring, thus making it challenging to effectively extract feature points for stable tracking. In tests 3 and 5, DynaSLAM performs better, which may be attributed to its ability to effectively identify and process dynamic feature points within the range of near-point extraction. In contrast, other algorithms do not distinguish between near and far points, leading to the inclusion of unstable distant points in tracking, thus affecting the system’s accuracy. In summary, GY-SLAM demonstrates superior accuracy and robustness in diverse motion modes, scene textures, and dynamism levels, consistently outperforming ORB-SLAM3 in all sequences and exceeding DynaSLAM in most data sequences.

**Figure 8:**
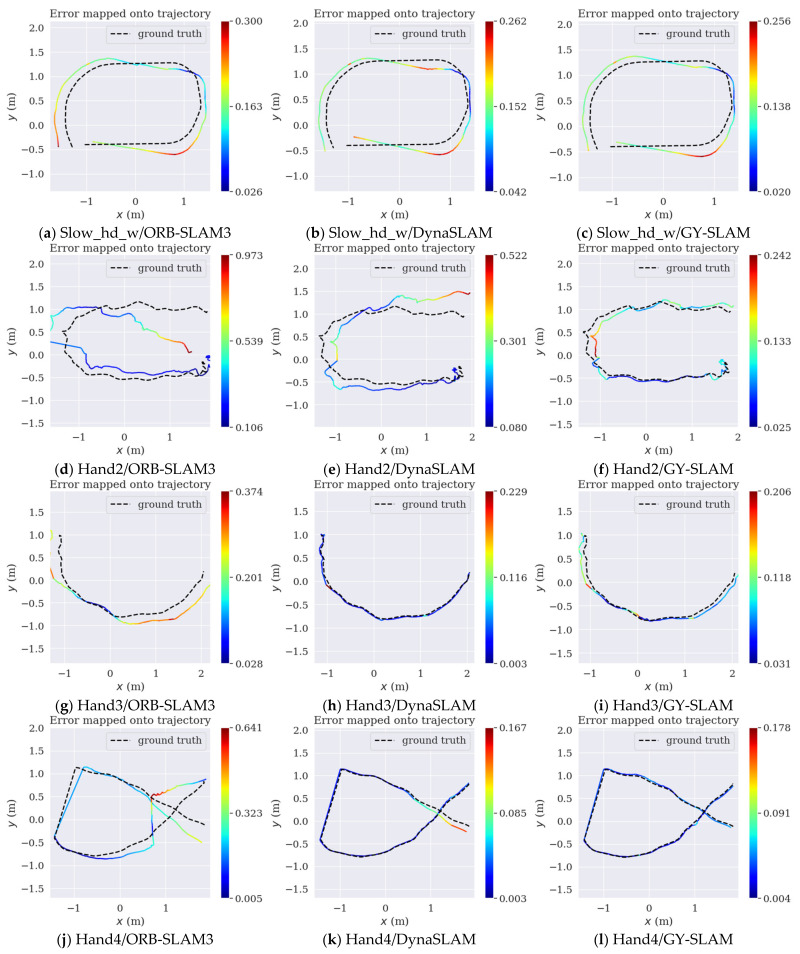
ATE graph evaluated by EVO. (**a**) Images (**a**–**c**), respectively, represent the ATE graphs of ORB-SLAM3, DynaSLAM, and GY-SLAM on the Slow_hd_w sequence; (**b**) Images (**d**–**f**) represent the ATE graphs of the three algorithms on the Hand2 sequence; (**c**) Images (**g**–**i**) represent the ATE graphs of the three algorithms on the Hand3 sequence; (**d**) Images (**j**–**l**) represent the ATE graphs of the three algorithms on the Hand4 sequence.

According to Table 12, GY-SLAM has significantly improved the system’s RMSE and Mean in terms of ATE, with the improvements reaching 85.1046% and 85.3191%, respectively. In test 3, where a handheld camera was used to continuously capture fast-moving people and robots at close range, DynaSLAM exhibited the best performance, reaffirming its advantage in distinguishing between near and far points. However, GY-SLAM demonstrates higher accuracy and robustness in medium to large dynamic scenes. These results indicate that GY-SLAM is competitive with the advanced SLAM algorithms in our dataset. The ATE graphs obtained by evaluating different algorithms using EVO on partial sequences of our custom dataset are shown in Figure 8.

#### 5.2.3. Tracking Time Evaluation

In practical applications, time efficiency is a crucial metric for evaluating the quality of SLAM systems. A time consumption experiment for various algorithms using the ‘Fr3_w_rpy’ sequence from the TUM RGB-D dataset is conducted. During this experiment, the average time taken by different algorithms to track a single frame is measured, as well as the time consumed during various key stages of the tracking process. The results are shown in Table 13, with time units in milliseconds.

The results in Table 13 prove that GY-SLAM achieves real-time processing, with each stage consuming less than 10 ms. Compared to GY-SLAM (*), the lightweight GY model brings a 41.5944% increase in detection speed and a 17.7975% improvement in SLAM operation speed. Although GY-SLAM takes an additional average of 5.3102 ms per frame compared to ORB-SLAM3, it significantly enhances the system’s accuracy and robustness in dynamic scenes.

#### 5.2.4. Efficacy of Feature Extraction and Mapping

**Figure 9:**
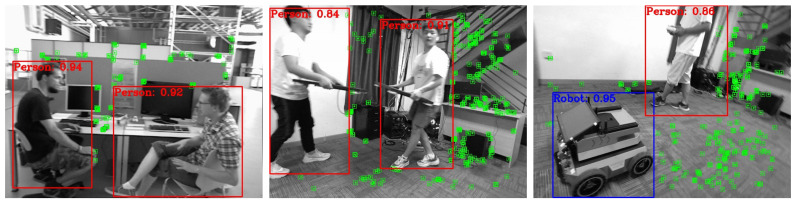
The feature extraction effects of GY-SLAM on different datasets. The green boxes represent the ORB feature points extracted by the SLAM system.

The ORB feature extraction effects of GY-SLAM on different datasets are illustrated in Figure 9.

**Figure 10:**
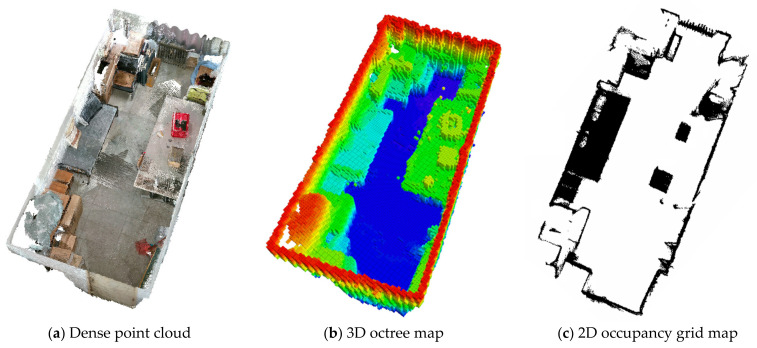
Efficacy of mapping. (**a**) The foundational purely static dense point cloud constructed by GY-SLAM; (**b**) The 3D octree map generated from the dense point cloud. Color is positively correlated with depth; (**c**) The 2D occupancy grid map generated from the dense point cloud.

We set the lower and upper projection limits of the occupancy grid map based on the robot chassis obstacle-clearance height and the overall height during the transportation of vegetable packages. To prevent any contact, we further raised the height limit by 0.1 m above these established limits. The purely static dense point cloud, 3D octree map, and 2D occupancy grid map constructed by GY-SLAM are illustrated in Figure 10.

## 6. Discussion

The performance of the GY-SLAM system in this study showcases the advancements in visual SLAM technology in dynamic environments. Our research emphasizes the importance of integrating VSLAM systems with deep learning in dynamic scenes, and the experimental results reveal limitations of GY-SLAM compared to DynaSLAM and DS-SLAM in processing near-field dynamic targets, targets predefined as static but actually in motion, and targets predefined as dynamic but stationary. These findings provide crucial directions for future research. We recommend that future studies consider integrating deep learning with geometric information to enhance the system’s ability to judge the motion state of targets and explore new strategies for distinguishing between near and far points to adapt to scenes of varying scales. Both of these approaches would further improve the accuracy and robustness of VSLAM systems.

In a broader context, this study highlights the application potential of VSLAM technology in the field of automated intelligent logistics. The improvements in the GY-SLAM system are not only crucial for enhancing the performance of robots in plant factory transportation environments, but they are also likely to have a positive impact on technological innovation in the logistics industry. We firmly believe that by integrating target detection technology, future VSLAM systems will be better adapted to complex and variable real-world application environments, making significant contributions to the advancement of automation technologies.

## 7. Conclusions

This study introduces a novel SLAM system, GY-SLAM, designed to enhance the localization, target detection, and mapping capabilities of robots in dynamic plant factory transportation environments. GY-SLAM extends ORB-SLAM3 by adding a target detection thread, a dense mapping thread, and a dynamic feature elimination module. In the target detection thread, GY-SLAM utilizes the GY target detection network, which is based on YOLOv5 and integrates GhostNet lightweight technology, CoordConv coordinate convolution, the CARAFE up-sampling operator, and the SE attention mechanism. These enhancements not only improve the model’s detection accuracy and generalization capability but also notably reduce the model’s complexity. While improving mAP@0.5 by 0.514%, the model simultaneously reduces parameters by 43.976%, computation by 46.488%, and weight by 41.752%. In the dense mapping thread, GY-SLAM utilizes dense point cloud data collected by depth cameras. After undergoing statistical filtering for noise reduction and voxel down-sampling, it can construct a dense point cloud for navigation, along with the corresponding 3D octree map and 2D occupancy grid map.

Performance evaluations on the TUM RGB-D and our proprietary dataset indicate that GY-SLAM exhibits significant improvements in dynamic environments compared to ORB-SLAM3, especially in handling high-dynamic scenes. It shows a remarkable 92.58% improvement in RMSE for ATE. Compared to YOLOv5s, the GY model brings a 41.5944% improvement in detection speed and a 17.7975% increase in SLAM operation speed to the system. In comparison with the current state-of-the-art DynaSLAM and DS-SLAM systems, GY-SLAM demonstrates superior performance in some dynamic sequences. However, we also noticed that GY-SLAM sometimes underperforms DynaSLAM and DS-SLAM in low-dynamic sequences and in processing near-field targets. In the future, we plan to integrate deep learning and geometric information to more accurately process dynamic feature points on all targets, while simultaneously improving strategies for distinguishing near and far points to further optimize GY-SLAM. Our long-term goal is to integrate GY-SLAM into the plant factory transportation robot, enabling it to support advanced tasks such as recognition, transportation, and route planning, thereby contributing to technological innovation in the logistics industry.

## Figures and Tables

**Table 1:** The experimental environment configurations.

Configuration	Parameter	Server Configuration
Hardware	CPU	AMD Ryzen 9 5900X 12-Core Processor (AMD, Luoyang, China)
	GPU	NVIDIA GeForce RTX 3060-12 GB (NVIDIA, Santa Clara, CA, USA)
	RAM	32 GB
Software	System	Windows 10/Ubuntu 18.04
	Python	3.9.18/2.7.17
Environment	PyTorch	1.12.1/1.9.0
	CUDA	11.6/11.1
	CuDNN	8.2.1/8.0.5

**Table 2:** The hyperparameter configuration of GY network training.

Hyperparameter	Value	Hyperparameter	Value
Epoch	300	Weight_decay	0.0005
Batch size	16	box	0.05
Lr0	0.01	cls	0.5
Lrf	0.1	obj	1.0
Momentum	0.937	Iou_t	0.20

**Table 3:** Equipment parameters for collecting the SLAM dataset.

Device	Parameter	Value
D455 Camera	Image Resolution	640 × 480 at 30 FPS (OV9782)
	FOV	86° × 57°
MR600 Robot	Overall Dimension	625 × 590 × 465 mm^3^
	Installation Heigh	350 mm
	Elevation Angle	10°
	Slow Speed	0.4 m/s
	Fast Speed	0.8 m/s
NOKOV	Marker	Φ15 mm × 10
Mars 2H	Camera Number	12
Cameras	3D Accuracy	±0.15 mm

**Table 4:** Lightweight network comparative experiment.

Network	mAP@0.5/%	Parameters	GFLOPs	Weight/M
CSPDarkNet53 (YOLOv5s)	94.850	7,018,216	15.774	13.70
ShuffleNetV2—YOLOv5s	89.949	3,794,120	7.989	7.68
MobileNetV3—YOLOv5s	91.358	3,543,926	6.297	7.17
GhostNet—YOLOv5s (GY*)	94.181	3,681,120	8.046	7.49

GY*: the model in its solely lightweight form, without any enhancements.

**Table 5:** Ablation experiment.

Test	CoordConv	CARAFE	SENet	GhostNet	mAP@0.5/%	Parameters	GFLOPs	Weight/M
1	×	×	×	×	94.850	7,018,216	15.774	13.70
2 (GY*)	×	×	×	√	94.181	3,681,120	8.046	7.49
3	√	×	×	√	95.153	3,759,008	8.144	7.64
4	×	√	×	√	95.220	3,821,224	8.315	7.77
5	√	√	×	√	95.317	3,899,112	8.414	7.92
6	×	×	√	√	94.872	3,713,888	8.073	7.56
7	√	×	√	√	95.238	3,791,776	8.171	7.70
8 (GY)	√	√	√	√	95.364	3,931,880	8.441	7.98

“×” means that the operation is not performed in the network. “√” means that the operation is performed in the network.

**Table 6:** Attention mechanism comparative experiment.

Attention	mAP@0.5/%	Parameters	GFLOPs	Weight/M
GY*	94.181	3,681,120	8.046	7.49
GY*-SE	94.872	3,713,888	8.073	7.56
GY*-CBAM	93.965	3,713,986	8.099	7.56
GY*-CA	94.645	3,706,768	8.074	7.55
GY*-ECA	94.148	3,681,123	8.048	7.49
GY*-EMA	94.230	3,722,336	8.340	7.57

**Table 7:** Algorithm comparative experiment.

Algorithm	mAP@0.5/%	Weight/M
YOLOv3	94.456	117.00
YOLOv5n	93.366	3.74
YOLOv5s	94.850	13.70
YOLOv5m	95.881	40.20
YOLOv5l	95.813	88.50
YOLOv5x	95.996	165.00
Ours (GY)	95.364	7.98

**Table 8:** Results of metric absolute trajectory error (ATE).

TUM RGB-D	ORB-SLAM3		DynaSLAM		DS-SLAM		GY-SLAM (Ours)		Improvements	
Sequences	RMSE	Mean	RMSE	Mean	RMSE	Mean	RMSE	Mean	RMSE/%	Mean/%
Fr3_s_hs	0.0566	0.0531	**0.0310**	**0.0263**	-	-	0.0326	0.0264	42.4028	50.2825
Fr3_s_static	0.0104	0.0093	0.0078	0.0069	**0.0065**	**0.0055**	0.0086	0.0075	17.3077	19.3548
Fr3_w_hs	0.2798	0.2376	0.0291	0.0259	0.0303	0.0258	**0.0268**	**0.0236**	90.4217	90.0673
Fr3_w_rpy	0.7203	0.6092	0.0548	0.0446	0.4442	0.3768	**0.0534**	**0.0384**	92.5864	93.6967
Fr3_w_static	0.0361	0.0284	0.0104	0.0091	**0.0081**	**0.0073**	0.0105	0.0094	70.9141	66.9014
Fr3_w_xyz	0.3725	0.3019	0.0311	0.0264	**0.0247**	**0.0186**	0.0292	0.0243	92.1611	91.9510

“-”: the symbol indicates that the set of data is missing. The bold font indicates that the indicator is the best of all algorithms.

**Table 9:** Results of metric translational drift (RPE).

TUM RGB-D	ORB-SLAM3		DynaSLAM		DS-SLAM		GY-SLAM (Ours)		Improvements	
Sequences	RMSE	Mean	RMSE	Mean	RMSE	Mean	RMSE	Mean	RMSE/%	Mean/%
Fr3_s_hs	0.0823	0.0658	**0.0485**	0.0419	-	-	0.0486	**0.0411**	40.9478	37.5380
Fr3_s_static	0.0159	0.0140	0.0112	0.0100	**0.0078**	**0.0068**	0.0123	0.0107	22.6415	23.5714
Fr3_w_hs	0.4186	0.3230	0.0422	0.0379	**0.0297**	**0.0256**	0.0393	0.0350	90.6116	89.1641
Fr3_w_rpy	1.0827	0.8892	0.0777	0.0641	0.1503	0.0942	**0.0746**	**0.0566**	93.1098	93.6347
Fr3_w_static	0.0551	0.0412	0.0166	0.0146	**0.0102**	**0.0091**	0.0160	0.0142	70.9619	65.5340
Fr3_w_xyz	0.5335	0.4003	0.0443	0.0384	**0.0333**	**0.0238**	0.0415	0.0362	92.2212	90.9568

**Table 10:** Results of metric rotational drift (RPE).

TUM RGB-D	ORB-SLAM3		DynaSLAM		DS-SLAM		GY-SLAM (Ours)		Improvements	
Sequences	RMSE	Mean	RMSE	Mean	RMSE	Mean	RMSE	Mean	RMSE/%	Mean/%
Fr3_s_hs	2.1441	1.8132	1.0404	0.9381	-	-	**1.0275**	**0.9218**	52.0778	49.1617
Fr3_s_static	0.4062	0.3657	0.3494	0.3152	**0.2735**	**0.2450**	0.3429	0.3043	15.5835	16.7897
Fr3_w_hs	9.2855	7.1467	1.0462	0.9543	**0.8142**	**0.7033**	1.0393	0.9282	88.8073	87.0122
Fr3_w_rpy	20.0856	15.7122	1.4833	**1.1780**	3.0042	1.9187	**1.4826**	1.2572	92.6186	91.9986
Fr3_w_static	0.9887	0.7647	0.3070	0.2789	**0.2690**	**0.2416**	0.3577	0.3201	63.8212	58.1404
Fr3_w_xyz	9.8547	7.1101	0.7542	0.6201	0.8266	0.5836	**0.7008**	**0.5635**	92.8887	92.0747

**Table 11:** Absolute trajectory error (ATE) results on Wheeled Dataset.

Test	Wheeled	ORB-SLAM3		DynaSLAM		GY-SLAM (Ours)		Improvements	
	Sequence	RMSE	Mean	RMSE	Mean	RMSE	Mean	RMSE/%	Mean/%
1	Mid_hd	0.0238	0.0193	0.0249	0.0212	**0.0224**	**0.0178**	6.0391	8.0776
2	Mid_ld_r	0.1714	0.1609	0.1919	0.1690	**0.1505**	**0.1354**	12.1878	15.8675
3	Mid_ld_rr	0.2019	0.1912	**0.1671**	**0.1546**	0.1832	0.1727	9.2641	9.6695
4	Slow_ld	0.1848	0.1548	0.1819	0.1524	**0.1739**	**0.1429**	5.8898	7.6930
5	Slow_hd	0.3166	0.3068	**0.2153**	**0.2077**	0.2277	0.2195	28.0829	28.4339
6	Slow_hd_w	0.1960	0.1875	0.1768	0.1714	**0.1482**	**0.1432**	24.4045	23.6476

**Table 12:** Absolute trajectory error (ATE) results on Handheld Dataset.

Test	Handheld	ORB-SLAM3		DynaSLAM		GY-SLAM (Ours)		Improvements	
	Sequence	RMSE	Mean	RMSE	Mean	RMSE	Mean	RMSE/%	Mean/%
1	Hand1	0.2203	0.2048	0.2113	0.1998	**0.1272**	**0.1092**	42.2582	46.6652
2	Hand2	0.3537	0.2943	0.2057	0.1787	**0.1059**	**0.0983**	70.0452	66.5881
3	Hand3	0.2386	0.2281	**0.0367**	**0.0312**	0.0913	0.0862	61.7216	62.2020
4	Hand4	0.2557	0.2172	0.0432	0.0319	**0.0381**	**0.0318**	85.1046	85.3191

**Table 13:** Time consumption costs of the Tracking thread.

Phase	ORB-SLAM3	DynaSLAM	GY-SLAM (*)	GY-SLAM (Ours)
Segmentation/Detection	×	979.3763	10.1302	5.9166
Feature Extraction	8.1198	23.4962	8.9929	8.9754
Light Track	×	1.2840	×	×
Geometric Correction	×	116.9251	×	×
Track	5.6370	3.5474	4.1580	4.0961
Total	14.5976	1251.2515	24.2180	19.9078

GY-SLAM (*), in which YOLOv5s is used instead of the GY model for target detection. “×” indicates that the algorithm does not have this function.

## Data Availability

The datasets used in this are the publicly available TUM RGB-D dataset and the publicly available Open Images dataset. They can be downloaded at the following links: 1. TUM RGB-D dataset (https://cvg.cit.tum.de/data/datasets, accessed on 28 November 2023); 2. Open Images dataset (https://storage.googleapis.com/openimages/web/index.html, accessed on 11 September 2023).
